# Design of a *trans *protease lentiviral packaging system that produces high titer virus

**DOI:** 10.1186/1742-4690-4-96

**Published:** 2007-12-28

**Authors:** Karen A Westerman, Zhujun Ao, Éric A Cohen, Philippe Leboulch

**Affiliations:** 1 Brigham and Women's Hospital, Department of Anesthesia (SR157), 75 Francis Street, Boston, MA, 02115, USA; 2 Institut de Recherches Cliniques de Montréal and Department of Microbiology and Immunology, Université of Montréal, Quebec, Canada; 3 Genetics Division, Department of Medicine and Harvard Medical School, Brigham and Women's Hospital, Harvard New Research Building, Boston, MA, 02115, USA

## Abstract

**Background:**

The structural and enzymatic proteins of the human immunodeficiency virus (HIV) are initially generated as two long polyproteins encoded from overlapping reading frames, one producing the structural proteins (Gag) and the second producing both structural and enzymatic proteins (Gag-Pol). The Gag to Gag-Pol ratio is critical for the proper assembly and maturation of viral particles. To minimize the risk of producing a replication competent lentivirus (RCL), we developed a "super-split" lentiviral packaging system in which Gag was separated from Pol with minimal loss of transducibility by supplying protease (PR) *in trans *independently of both Gag and Pol.

**Results:**

In developing this "super-split" packaging system, we incorporated several new safety features that include removing the Gag/Gag-Pol frameshift, splitting the Gag, PR, and reverse transcriptase/integrase (RT/IN) functions onto separate plasmids, and greatly reducing the nucleotide sequence overlap between vector and Gag and between Gag and Pol. As part of the construction of this novel system, we used a truncated form of the accessory protein Vpr, which binds the P6 region of Gag, as a vehicle to deliver both PR and RT/IN as fusion proteins to the site of viral assembly and budding. We also replaced *wt *PR with a slightly less active T26S PR mutant in an effort to prevent premature processing and cytoxicity associated with *wt *PR. This novel "super-split" packaging system yielded lentiviral titers comparable to those generated by conventional lentiviral packaging where Gag-Pol is supplied intact (1.0 × 10^6 ^TU/ml, unconcentrated).

**Conclusion:**

Here, we were able to create a true "split-function" lentiviral packaging system that has the potential to be used for gene therapy applications. This novel system incorporates many new safety features while maintaining high titers. In addition, because PR is supplied *in trans*, this unique system may also provide opportunities to examine viral protein processing and maturation.

## Background

The genome of Human Immunodeficiency Virus Type 1 (HIV-1) is complex in that it employs overlapping reading frames to encode two essential polyproteins known as Gag and Gag-Pol. The Gag polyprotein precursor supplies the structural components of the virus that include the matrix (MAp17), capsid (CAp17), nucleocapsid (NCp7), and p6 proteins while the Pol polyprotein precursor supplies the viral enzymes protease (PR, p11), reverse transcriptase/Rnase H (RT, p66/p51), and integrase (IN, p32) (for review see [1,2]). The concentrations of Gag to Gag-Pol polyproteins are maintained at a ratio of 20:1 through a frameshift mechanism in which the ribosome slips by -1 on a heptanucleotide AU rich sequence located at the end of the NCp7 protein [3]. The ensuing frameshift results in the ribosome reading through P6 to produce the full length Gag-Pol polyprotein. This 20:1 ratio of the Gag to Gag-Pol has been shown by many researchers to be critical for the production of "infectious" viral particles. Attempts to vary the 20:1 polyprotein ratio, has resulted in decreases in virus infectivity and stability [4-6]. In addition, the expression of Gag without Gag-Pol has been shown to result in the assembly of particles that are non-infectious [7], and in the reverse case, when Gag-Pol is expressed without Gag, there is efficient PR processing but no production of virions [8].

PR is essential for the processing of the viral polyprotein precursors and thus plays an important role in the maturation of viral particles and in the production of infectious particles [9-12]. During the assembly of the Gag and Gag-Pol polyproteins, PR is initially inactive. As the concentration of polyproteins increases and the virion components are confined in the budding particle, PR then dimerizes and becomes active [13-16]. Once PR is active, it then sequentially cleaves the assembled precursor polyproteins resulting in the transformation of the immature viral particle into a mature infectious virion [10,12]. Hence, the correct balance of Gag to Gag-Pol is critical to ensure that not only the viral enzymes are incorporated into the viral particles but also that PR becomes activated at the appropriate time to prevent the production of defective particles with reduced infectivity due to premature processing of the Gag polyproteins [9,14,17].

Here we describe a novel lentiviral packaging system in which not only is Gag supplied separately from Pol, but PR is also supplied independently. One of the greatest concerns with the construction of retroviral and lentiviral packaging systems is the production of RCR (replication competent retrovirus) and RCL (replication competent lentivirus), respectively. As the production of RCR/RCL is believed to occur through homologous recombination between overlapping sequences, researchers have minimized this risk by dividing the functional components of the viral genomes onto separate expression plasmids. In the case of retroviruses, the vector, GagPol, and envelope have all been supplied separately in what was called a "split-function" packaging system [18]. In the case of lentiviruses, which are more complex, it was found that not only can the Gag-Pol be separated from the vector and envelope, but that the accessory proteins (Vif, Vpr, Vpu, and Nef) and regulatory proteins (Rev and Tat) could also be either eliminated or supplied *in trans *[19-21]. The reasoning behind these split-function retroviral and lentiviral packaging systems is that it is much less likely that 2, 3, or even 4 recombinations would occur to generate a RCR/RCL, which in turn makes these split-function systems inherently safer. This is especially important for large-scale, clinical grade, vector production. In the case of lentiviral packaging systems, no RCL events have been detected to date, probably because the vesicular stomatitis virus glycoprotein G (VSV-G), which is widely used as pseudotyping envelope and is cytotoxic when constitutively expressed, makes it difficult to form a *bona fide *RCL that comprises and expresses the VSV-G gene. However RCRs have been detected in split-function retroviral packaging lines that make use of ecotropic or amphotropic retroviral envelopes [22,23]. In view of the highly pathogenic nature of HIV-1, it is thus of the utmost importance to ensure that the safest possible lentiviral packaging systems are used for gene therapy applications to prevent the slightest possibility of RCL or even pre-RCL formation. Here we have devised a "super-split" 7-plasmid lentiviral packaging system with minimal loss of transducibility with which more than 4 recombination events would be required to produce a viable RCL.

A key feature of this system is the use of the p6-binding domain of the accessory HIV protein Vpr to tether fusion proteins to the budding virions, an approach pioneered by Kappes' and Hahn's groups [24-26] and ourselves [27,28]. In the past, we (unpublished data) as well as Wu, et al. [29] have designed split-function lentiviral packaging systems in which Gag-PR was supplied separately from RT-IN by means of Vpr-mediated tethering. However, these previous attempts either resulted in a substantial decrease in lentiviral titers or did not comprise a true split of the Gag-Pol gene. In the latter case, a stop codon was introduced at the start of RT and IN to prevent the expression of RT and IN, so that RT and IN sequences remained present in the Gag-PR expression plasmid [29]. This configuration retains a residual risk of RCL formation by sequence read-through, reversion or recombination. Here, we have improved upon these systems by creating a true split-function lentiviral packaging system in which Gag, PR, and RT/IN are supplied by three independent plasmids. This "super-split" system affords an additional level of protection against RCL formation through a higher level of true plasmid separation while unexpectedly restoring useful lentiviral titers.

## Results

### Delivering the Pol proteins in trans to the viral particles

**Figure 1:**
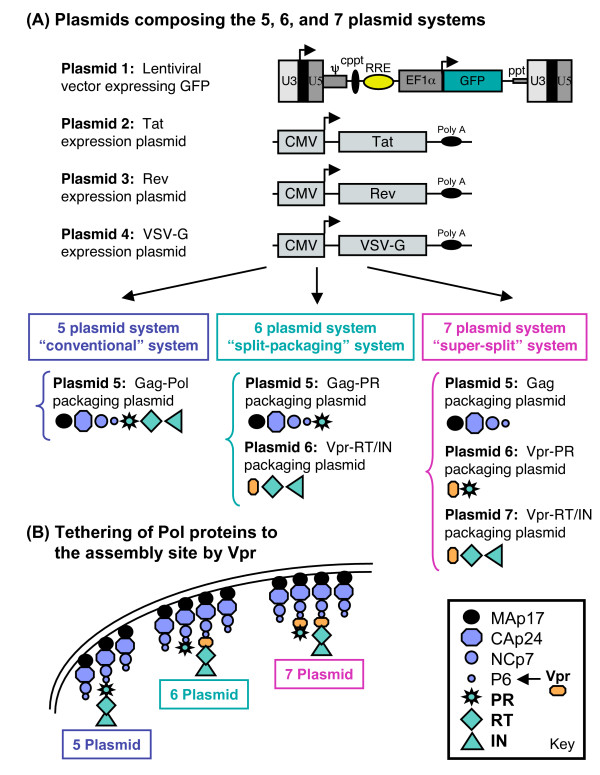
**Schematic of the components involved in the 5, 6, and 7 plasmid systems**. **(A) **Diagram of the 4 plasmids used in common for all three packaging systems for the production of virus, followed by a brief description of the packaging plasmids used for each of the corresponding systems (more detail is shown in Fig. 2). **(B) **Schematic depicting the assembly site of the viral proteins as it takes place in the 5 plasmid system, here the Gag and Gag-Pol precursor proteins are targeted to the cell membrane through the membrane-binding domain located at the N-terminus of MAp17, and the assembly sites of the 6 and 7 plasmid systems where the Gag proteins are targeted to cell membrane by MAp17, and the Pol proteins (PR and RT/IN) are targeted through tethering of Vpr to P6.

**Figure 2:**
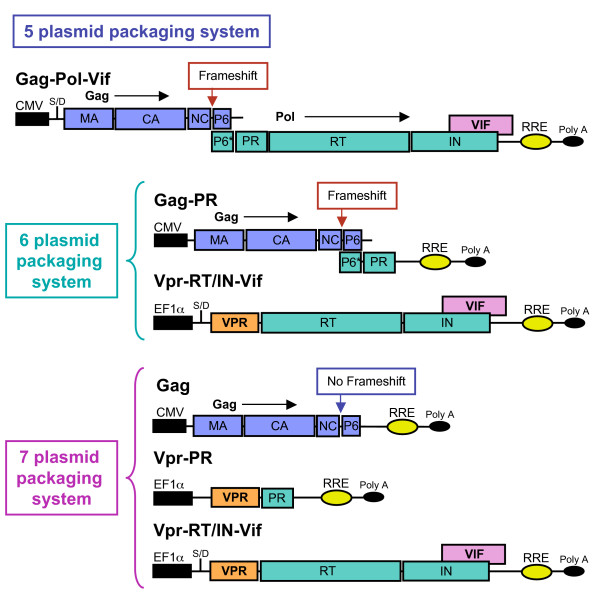
**Schematic showing the packaging plasmids used in the 5, 6, and 7 plasmid systems**. Gag proteins are represented in blue, the Pol proteins in green, Vpr in orange, and Vif in pink. The packaging plasmid in the 5 plasmid system is located at the top of the diagram, only one plasmid is used to express both Gag and Gag-Pol (after frameshifting). The packaging plasmids in the 6 plasmid system are located in the middle of the diagram, two plasmids are used, one that expresses Gag and Gag-PR (after frameshifting) and the other expressing Vpr-RT/IN-Vif (reverse transcriptase, integrase, and Vif). The packaging plasmids in the 7 plasmid system are located at the bottom of the diagram, three plasmids are used, the first expressing Gag alone (there is no frame shift), and the second and third plasmids expressing the Pol components, Vpr-PR (protease alone) and Vpr-RT/IN-Vif (reverse transcriptase, integrase, and Vif), respectively.

During the viral life cycle, the Gag (Pr55Gag) and Gag-Pol (Pr160Gag-Pol) precursor polyproteins are targeted to the cell membrane for assembly via the membrane-binding domain (M), which consists of a N-terminal myristylic acid group and a highly basic stretch of amino-acids at the N terminus of MAp17 protein [30-33]. The first step in designing a split Gag-Pol packaging system is to consider how to deliver the Pol proteins, which are normally incorporated via the Gag-Pol precursor polyprotein, to the viral assembly site. Since Vpr can be efficiently incorporated into viral particles (approximately 200 molecules per virion) by an independent mechanism, that is, through an interaction with the C-terminal of P6 on the Gag precursor polyprotein [34-36], we chose to use Vpr to supply the Pol proteins (PR and RT/IN) independently. A truncated form of Vpr (1–88) was selected since it has the ability to be packaged in HIV particles as efficiently as wild type Vpr but is strongly defective in its ability to induce a G2 cell cycle arrest [37]. A representation of Vpr tethering of the Pol components supplied *in trans *to the viral assembly site is shown in (Fig. 1B), while the packaging plasmids for each of the 3 lentiviral systems presented here are shown in (Fig. 2).

### Structure of the three lentiviral packaging systems

The data presented here compares 3 different lentiviral packaging systems. The first, referred to as the **"5 plasmid system"**, is a conventional lentiviral packaging system where Gag-Pol is supplied from a single expression plasmid. In addition to the packaging plasmid, which contains both Gag-Pol and Vif (Vpr, Vpu, Tat, Rev, ENV, and Nef were all deleted), four other expression plasmids are used to generate virus: the first contains the lentiviral vector that encodes GFP, the second expresses Tat, the third Rev, and the fourth VSV-G. The second system, referred to as the **"6 plasmid system"**, is a split-packaging system in which the Gag-Pol functions are expressed by two separate plasmids, one for Gag-PR and the other for RT-IN. The Gag-PR expression plasmid was derived from the aforementioned Gag-Pol plasmid in which all the RT, IN, and Vif sequences were deleted. The second packaging plasmid consists of Vpr fused to RT/IN-Vif, a splice donor site to allow for the proper splicing and expression of Vif, and the natural PR cleavage site for RT (33 bases before the start of RT) to allow for proper PR processing of the RT and IN proteins. The third system, referred to as the **"7 plasmid system"**, is a "super-split" packaging system in which the functional components of the Gag-Pol are expressed from three separate plasmids. The first plasmid contains only the Gag gene from which the frameshift has been mutated and all the regions that encode the Pol proteins deleted. The second plasmid contains PR fused to Vpr along with the natural PR cleavage site (15 bases before the start of PR). The third plasmid is the same Vpr-RT/IN-Vif fusion plasmid used for the 6 plasmid system. Diagrams of the plasmids used for all three packaging systems are shown in (Figs. 1 and 2).

### Titer analysis of the 5, 6, and 7 plasmid systems

Optimizing parameters, such as molar ratios of one plasmid to another, as well as comparing one system to another, were performed by means of a *wt*-LTR lentiviral vector that expresses GFP driven by an EF1α promoter. Since the 6 and 7 plasmid systems described here are not conventional, we suspected that p24 and RT assays may not accurately reflect viral titers. The p24 assay gives information about the amount of CAp24 present but does not discriminate infectious from non-infectious particles. In the same respect, the RT assay gives information on RT activity, but it may be difficult to interpret as the 6 and 7 plasmid systems supply RT *in trans*. We thus chose instead to measure functional infectious viral titers by scoring stable GFP expression in target cells upon chromosomal integration of the provirus. These titers were determined by transfecting 293T cells with 5, 6, or 7 plasmids, collecting the supernatants 48 h later, transducing NIH 3T3 and Jurkat cells with varying amounts of these viral supernatants, and then monitoring the transduced NIH 3T3 and Jurkat cells for the production of GFP by FACS.

### Results from the split-packaging 6 plasmid system

**Figure 3:**
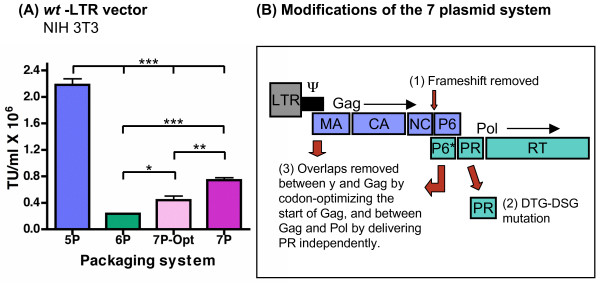
**The 7 plasmid system: functional titers and modifications**. **(A) **Functional titers were obtained using a *wt*-LTR lentiviral vector containing green fluorescent protein (GFP) driven by an EF1α promoter. NIH 3T3 cells were infected with serial dilutions of viral supernatants produced by the 5, 6, or 7 plasmid systems as mentioned in the Methods. The number of transducing units (TU) was determined by multiplying the number of cells plated by the percentage of GFP positive cells (determined by FACS) by the dilution factor. The mean titer for the 5 plasmid system, shown in blue, was 2.2 × 10^6 ^TU/ml, for the 6 plasmid system, shown in green, 2.4 × 10^5 ^TU/ml, for the 7 plasmid system with the optimized Gag, shown in light pink, 4.4 × 10^5 ^TU/ml, and for the 7 plasmid system, shown in dark pink, 7.4 × 10^5 ^TU/ml. Error bars represent SEM, 5 independent experiments are represented (N = 5), * p = 0.03 (6P versus 7P-Opt), ** p = 0.003 (7P-Opt versus 7P), *** p < or = 0.0002 (6P versus 7P, 5P versus 6P, and 5P versus 7P-Opt, 5P versus 7P) as determined by unpaired t-test using Prism 4 software. **(B) **Schematic showing the safety modifications incorporated into the 7 plasmid system. Gag proteins are represented in blue and Pol proteins in green. (1) The Gag to Gag-Pol frameshift was eliminated (AA**T TT TTA **GGG became AA**C TTC TTA **GGG). (2) PR was expressed independently of both Gag and Pol. In addition, the active site of PR was changed from DTG to DSG to create the T26S mutant PR. (3) The sequences that overlapped between the packaging signal (Ψ) and Gag, and between Gag and Pol (at P6) were greatly reduced.

**Figure 4:**
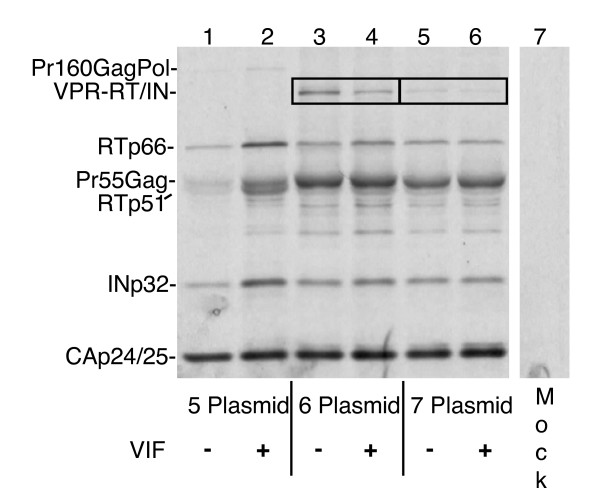
**Protein analysis of viral particles generated by the 5, 6, and 7 plasmid systems**. 293T cells were transfected with the 5 plasmid system (lanes 1 and 2), the 6 plasmid system (lanes 3 and 4), or the non-optimized 7 plasmid system (lanes 5 and 6), with (lanes 2, 4, 6) or without (lanes 1, 3, 5) Vif. Transfected cells were labeled with [^35^S] methionine for 12 h, 48 h post transfection. Radiolabeled viral particles were pelleted, lysed, immunoprecipitated with anti-HIV serum and analyzed on 12.5% SDS-PAGE. The position of viral proteins are indicated, the boxed region shows the location of Vpr-RT/IN in which less accumulation of unprocessed Vpr-RT/IN can be seen for the 7 plasmid system as compared to the 6 plasmid system. Quantitative analysis of the CAp24 and Pr55Gag bands showed a 3 fold decrease in ratio of CAp24 to Pr55Gag for the 6 plasmid system and a 2 fold decrease for the 7 plasmid system (Cap24/Pr55Gag; *5 plasmid system *6.1 and 4.3, *6 plasmid system *1.6 and 1.8, *7 plasmid system *2.6 and 2.1, without and with Vif respectively). Lanes are not loaded equally. Mock, uninfected.

The initial question in constructing the 6 plasmid system was how to best separate the Gag-Pol polyprotein precursor without affecting the processing of the viral particles. We decided that the safest location to separate the Gag-Pol was likely to be between PR and RT. There were two reasons for choosing this location. The first was to preserve the frameshift in order to minimize disturbing PR expression by maintaining the 20:1 ratio with Gag. The second was to avoid the 208 nucleotide overlap that occurs between the end of Gag and the start of Pol. To determine if the viral particles produced by this system would be infectious, 293T cells were transfected with either the 5 plasmid or 6 plasmid system and the resulting supernatants were used to transduce NIH 3T3 cells. Titers were then determined by FACS analysis for the expression of GFP. As shown in (Fig. 3A), titers obtained with the 6 plasmid system averaged 2.4 × 10^5 ^TU/ml whereas the titers obtained with the 5 plasmid system averaged 2.2 × 10^6 ^TU/ml. While these results indicate that the 6 plasmid produces infectious particles at respectable titers, the titers generated were consistently 9 times lower than those of the conventional 5 plasmid system. We hypothesized that the lower titers generated by the 6 plasmid system may be caused by less efficient processing of the precursor polyproteins as a result of splitting RT/IN from Gag-Pol. In order to determine if there was defective processing of viral polyproteins by the 6 plasmid system, we pelleted viral particles from culture supernatants and analyzed virion-associated protein products by immunoprecipitation using serum from an HIV positive patient. Results in Fig. 4 show that RT and IN are efficiently packaged into virions for the 6 plasmid system, with the levels of RT and IN to Gag (Pr55Gag and CAp24) comparable to those found in the conventional 5 plasmid system. This indicated that the fusion of Vpr with RT/IN was successful in delivering RT and IN to the virions. Next, we looked at the processing of the precursor polyproteins. We found that there was efficient processing of the virions produced by the 5 plasmid system with little accumulation of the Gag (Pr55Gag) or Gag-Pol (Pr160Gag-Pol) whereas virions produced by the 6 plasmid system showed a processing defect indicated by an accumulation of both Pr55Gag and Vpr-RT/IN (Fig. 4). Quantitative analysis by laser densitometry scanning of the CAp24 and Pr55Gag bands showed that the ratio of CAp24 (from processed Gag) to Pr55Gag (unprocessed precursor Gag) was 3-fold lower in the 6 plasmid system than in the 5 plasmid system (CAp24/Pr55Gag; *5 plasmid system *6.1 and 4.3, *6 plasmid system *1.6 and 1.8, without and with Vif respectively). Taken together, these results indicate that activation and release of PR were inefficient, and that the titers of the 6 plasmid system could possibly be rescued by increasing expression of PR.

### Titer rescue of the 6 plasmid system by supplying PR *in trans*

To correct the processing problem detected with the 6 plasmid system, we decided to express PR separately from Gag, resulting in the development of a "super-split" 7 plasmid system. Before constructing this new system, there were three areas of concern that needed to be addressed: (i) What to do with the frameshift, (ii) How to deliver PR *in trans *without cytotoxicity or loss of infectivity, and (iii) How to minimize the sequence overlap between the packaging signal and Gag, and between Gag and Pol, see (Fig. 3B).

In confronting the first concern, we decided to remove the frameshift in order to completely separate Gag from Pol. This was performed using PCR to generate a fragment, between the Nsi I site (found in the CAp24) and the Bgl II site (just after NCp7), which encompasses the area of frameshift at the end of NCp7. This frameshift sequence was changed from AA**T TTT TTA **GGG to AA**C TTC TTA **GGG. A second PCR was performed from the Bgl II site (just after NCp7) to the stop codon of P6 in order to eliminate PR. The result was a Gag expression plasmid in which both the frameshift and PR had been eliminated.

**Figure 5:**
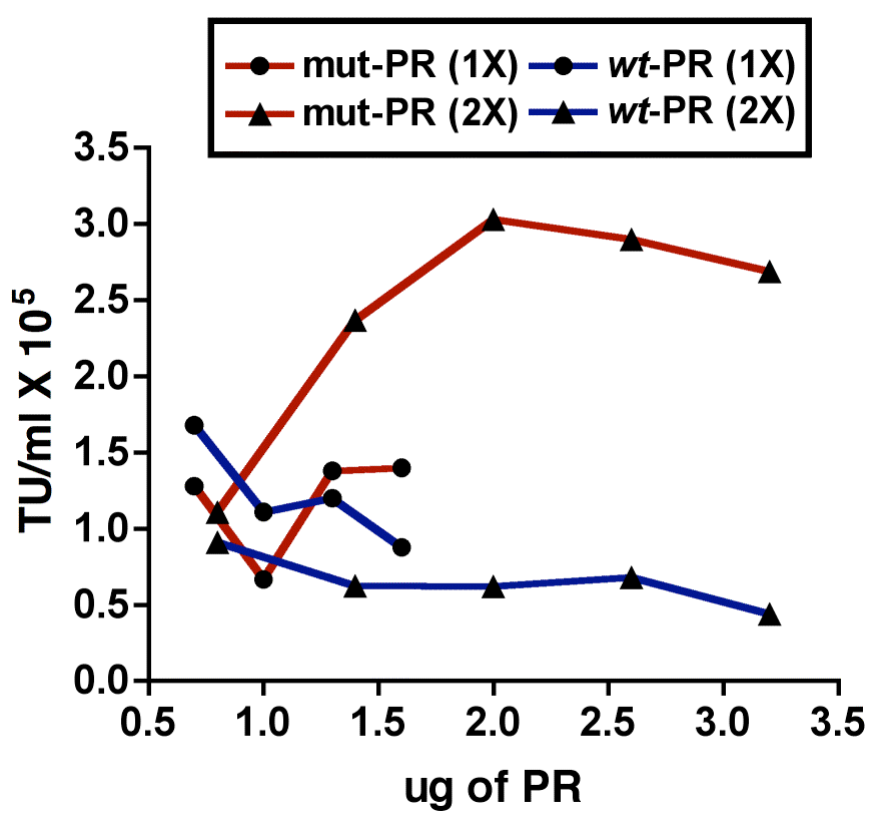
**Comparison of titers produced by PR expression plasmids: *wt *versus T26S mutant**. NIH 3T3 cells were infected with serial dilutions of viral supernatants produced by the 7 plasmid system with either the *wt *(blue) or T26S mutant (red) PR. Titers were determined by monitoring transduced NIH 3T3 cells for the production of GFP by FACS. In this study, the lentiviral vector (*wt*-LTR expressing GFP), Rev, Tat, VSV-G, Gag (non-optimized), and Vpr-RT/IN DNA amounts remained constant, while the DNA amounts of Vpr-*wt *PR (*wt *protease, shown in blue) and Vpr-T26S PR (mutant protease, shown in red) varied. Experiments were performed using two concentrations for Gag and Vpr-RT/IN: (1×) using 1.3 μg Gag and 2.3 μg Vpr-RT/IN DNA with varying amounts of PR DNA (0.7 μg, 1.0 μg, 1.3 μg, and 1.6 μg), indicated on the graph by circles (●), and (2×) using 2.6 μg Gag and 4.5 μg Vpr-RT/IN DNA along with varying amounts of PR DNA (0.8 μg, 1.4 μg, 2.0 μg 2.6 μg, 3.2 μg), indicated on the graph by triangles (▲). In these initial studies the Vpr-RT/IN plasmid did not contain Vif, the functional titers ranged from 0.4 × 10^5 ^TU/ml to 3.0 × 10^5 ^TU/ml. N = 1.

The next step was to determine how to express PR optimally. This was problematic in that PR is central to the processing of the precursor polyproteins and as a consequence to the maturation of the viral particles [9]. The main concern was that too much PR may be expressed resulting in premature processing and cytoxicity [9,14,17]. To address this concern, we expressed a less active PR mutant as an alternative to the *wt *PR. In searching the literature, we chose a PR mutant with an altered active site in which Asp-Thr-Gly was changed to Asp-Ser-Gly (T26S) [9,38,39]. This mutant was shown to have a slightly reduced protease activity (4–10 fold), with very little effect on viral assembly or maturation, and a markedly reduced cytotoxicity that may result from a shift in the pH needed for its activation [38,39]. The T26S mutation was included in the construction of the PR expression vector in which PR was fused to Vpr, leaving only 15 bases before PR for protease processing. To test whether there was an advantage in using the mutant form of PR, pilot studies were preformed to optimize viral titers by varying the concentrations of the Gag, PR, and RT/IN expression plasmids in order to compensate for molar differences of the plasmids used, as well as for differences in the activity of *wt *versus mutant protease. One of these pilot studies is shown in (Fig. 5). In this study 293T cells were transfected with the 7 plasmid system, in which the lentiviral GFP vector, Rev, Tat, VSV-G, Gag, and Vpr-RT/IN DNA amounts remained constant, while the concentrations of either the *wt *protease (Vpr-*wt *PR, shown in blue) or mutant protease (Vpr-T26S PR, shown in red) expression plasmids varied. As can be seen in (Fig. 5), the titers obtained when mutant or *wt *PR was delivered independently of Gag ranged from 0.4 × 10^5 ^TU/ml to 3.0 × 10^5 ^TU/ml, indicating that PR can be supplied *in trans *to produce infectious particles. In addition, when the T26S mutant PR was used, replacing the *wt *PR, we were able to obtain equivalent or higher (3 fold) titers than those obtained with the *wt *PR. We consequently continued to optimize DNA concentrations further improving viral titers produced with the 7 plasmid system, using the T26S mutant PR in place of the *wt *PR (Fig. 3A).

The third goal in constructing the 7 plasmid system, as seen in (Fig. 3B), was to minimize the sequence overlap between packaging signal and Gag and between Gag and Pol. The first overlap consisted of 542 bases and was minimized (from 542 to 55 bases) by optimizing the codons at the start of Gag, that is, by using alternate nucleotides for the codons while maintaining the originally encoded Gag amino acid sequence. The second overlap, located at the junction of Gag and Pol, was minimized in the two previous steps by removing the frameshift and separating Gag from PR (208 bases reduced to 54 bases). To determine whether the use of this optimized Gag had an impact on titers generated by the 7 plasmid system, we compared functional titers obtained with the original versus the Gag-optimized expression plasmids. Titer results for the 5 plasmid system, the 6 plasmid system, the 7 plasmid system after optimizing Gag, and the 7 plasmid system where Gag is not optimized, are shown in (Fig. 3A). These titers were obtained after optimizing transfections for variations in total DNA concentration and for molar differences in plasmids used to generate virus for the 6 (Gag-PR and Vpr-RT/IN-Vif) and 7 (Gag, Vpr-T26S PR, and Vpr-RT/IN-Vif) plasmid systems. As can be seen in (Fig. 3A), the 7 plasmid system in which PR is supplied independently of Gag and RT/IN generated titers that were about 2–3 fold higher than those obtained with the 6 plasmid system. Titers achieved with the 6 plasmid system averaged 2.4 × 10^5 ^TU/ml and were 9 fold lower than titers obtained with the 5 plasmid system, whereas titers obtained with the 7 plasmid system averaged 4.4 × 10^5 ^TU/ml with the optimized Gag, and 7.4 × 10^5 ^TU/ml with the non-optimized Gag, that is only about 3 to 5 fold lower than with the 5 plasmid system.

In addition to looking at the functional titers, we analyzed the viral particles generated by the 7 plasmid system to determine whether protein processing had improved by supplying PR independently of Gag. The results shown in (Fig. 4) demonstrate that the Vpr fusions are effective in supplying the Pol components *in trans *for both mutant PR and RT/IN. The virions produced by the 7 plasmid system, in which PR is delivered independently, showed more processed proteins (CAp24, RT, and IN) with less accumulation of both Pr55Gag and Vpr-RT/IN. Quantitative analysis of the CAp24 and Pr55Gag bands revealed that the ratio of CAp24 (CA from processed Gag) to Pr55Gag (unprocessed Gag precursor) had improved compared to the 6 plasmid system and was now just 2 fold lower than with the 5 plasmid system (Cap24/Pr55Gag; *5 plasmid system *6.1 and 4.3, *6 plasmid system *1.6 and 1.8, *7 plasmid system *2.6 and 2.1, without and with Vif respectively). In addition, because we generally saw a slight increase in titers in the presence of Vif (data not shown) we also looked at the processing in relation to the presence of Vif for all three systems. We were unable to establish conclusively that a change had occurred in the processing of the Gag precursor in the presence of Vif, although we detected an improvement in the processing of Vpr-RT/IN with the 6 plasmid system, as can be seen in Figure 4 by the concurrent reduction in Vpr-RT/IN and increase in RT (lanes 3 and 4).

### Self-inactivating (SIN) vector improves viral titers

**Figure 6:**
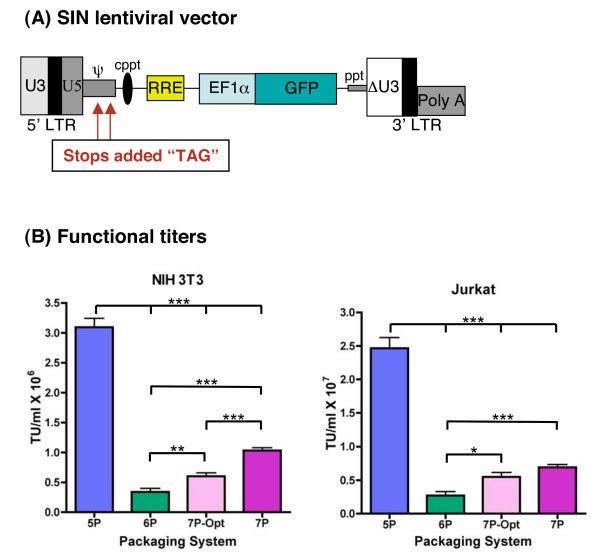
**Titer results for the 5, 6, and 7 plasmid systems with the SIN lentiviral vector**. **(A) **Diagram showing the structure of the SIN lentiviral vector which contains the following safety features: a 400 bp deletion in the U3 region of the 3' LTR, a complete deletion of the 3' LTR U5 region replaced by an ideal termination/polyadenylation sequence, and two stops placed within the packaging signal (Ψ) to prevent the production of unwanted transcripts. This vector also contains an unmodified 5' LTR, the central polypurine tract, RRE, and GFP driven by an EF1α promoter. **(B) **NIH3T3 and Jurkat cells were infected with serial dilutions of viral supernatants produced using a SIN lentiviral vector along with the 5, 6, or 7 plasmid packaging systems. Titers were determined by monitoring transduced cells for the production of GFP by FACS. For NIH3T3 cells the mean titer with the 5 plasmid system, shown in blue, was 3.1 × 10^6 ^TU/ml, the 6 plasmid system, shown in green, 3.4 × 10^5 ^TU/ml, and the 7 plasmid system with and without the optimized Gag, shown in light and dark pink, 6.0 × 10^5 ^TU/ml, and 1.0 × 10^6 ^TU/ml, respectively. ** p = 0.009 (6P versus 7P-Opt), *** p < or = 0.0002 (6P versus 7P, 7P-Opt versus 7P, 5P versus 6P, and 5P versus 7P-Opt, 5P versus 7P). For Jurkat cells the mean titer for the 5 plasmid system, shown in blue, was 2.5 × 10^7 ^TU/ml, the 6 plasmid system, shown in green, 2.7 × 10^6 ^TU/ml, the 7 plasmid system with and without the optimized Gag, shown in light and dark pink, 5.5 × 10^6 ^and 6.9 × 10^6 ^TU/ml, respectively. * p = 0.01 (6P versus 7P-Opt), *** p < 0.0001 (6P versus 7P, 5P versus 6P, and 5P versus 7P-Opt, 5P versus 7P). Error bars represent SEM, data represents 6 independent experiments (N = 6), statistical analysis was determined by unpaired t-test using Prism 4 software.

In addition to modifying the packaging system, we also constructed a SIN lentiviral vector to improve further the safety of the system by decreasing the risk of provirus mobilization and RCL formation. This SIN vector was constructed by modifying the U3 and U5 regions of the 3' LTR, as follows: a 400 bp deletion was created in the U3 region between the EcoRV to the Pvu II restriction sites to remove viral enhancer and promoter, and the U5 region was entirely eliminated and replaced by an "ideal" termination/polyadenylation sequence (ATG TGT GTG TTG GTT TTT TGT GT). In addition, two stop codons were also introduced within the region where the packaging signal and Gag overlap, so that Gag could not be reconstituted if a recombination occurred and to prevent the translation of a residual Gag peptide. The remaining portions of this vector are identical to those of the *wt*-LTR lentiviral vector, that is, they both contain an unmodified 5' LTR (so that the lentiviral vector remains Tat dependent), the central polypurine tract, RRE, and an Ef1α promoter driving GFP expression (Fig. 6A). In conjunction with the SIN vector we chose to continue to supply Tat *in trans *due to safety concerns, that is to say, since the 5' LTR in our vectors do not contain a strong promoter (such as CMV or RSV) and still require Tat to properly activate their HIV-1 promoter, than the Tat transactivation of the promoter acts as a safeguard preventing the production of full length packagable transcripts by the integrated vector. To determine if this SIN vector would significantly affect titers, 293T cells were transfected with plasmids composing each of the three packaging systems in conjunction with the SIN vector (Fig. 6B), the resulting supernatants were then used to transduce NIH 3T3 cells. Titers were determined by FACS analysis for the expression of GFP. For all three systems, titers improved by 1.4 fold when the SIN vector was used. The 5 plasmid system increased from 2.2 × 10^6 ^TU/ml to 3.1 × 10^6 ^TU/ml, the 6 plasmid system from 2.4 × 10^5 ^TU/ml to 3.4 × 10^5 ^TU/ml, the 7 plasmid system with the optimized Gag from 4.4 × 10^5 ^TU/ml to 6.0 × 10^5 ^TU/ml, and the 7 plasmid system with the non-optimized Gag from 7.4 × 10^5 ^TU/ml to 1.0 × 10^6 ^TU/ml. The increase in viral titers when the SIN vector was used was such that the 7 plasmid system provided functional titers (1.0 × 10^6 ^TU/ml) that were just 2 fold lower than those obtained when the *wt *lentiviral vector was used with the conventional 5 plasmid system (2.2 × 10^6 ^TU/ml).

To demonstrate that the 7 plasmid system is capable of efficiently transducing other cell types, such as human T cells, we also transduced Jurkat cells using the GFP SIN vector along with the 5, 6, and 7 plasmid systems. As shown in (Fig. 6B), titers obtained with the 6 plasmid system averaged 2.7 × 10^6 ^TU/ml, once again 9 fold lower than titers obtained with the 5 plasmid system, titers obtained for the 7 plasmid system averaged 5.5 × 10^6 ^TU/ml with the optimized Gag and 6.9 × 10^6 ^TU/ml with the non-optimized Gag, these titers were 2–3 times higher than those obtained with 6 plasmid system and just 4 times lower than those obtained using the 5 plasmid system.

## Discussion

Here we describe a novel "super-split" lentiviral packaging system in which the overlapping Gag and Pol polyprotein precursors are completely separated and supplied independently to produce high titer virus. This approach also brings further evidence that Vpr can be used as a vehicle to incorporate the Pol components, PR and RT/IN, effectively into viral particles, as we and others have successfully used Vpr fusions to supply proteins *in trans *to viral particles [24-28]. Vpr has also been used to supply RT/IN as part of a safer lentiviral packaging system in which Gag-PR and RT/IN functions were delivered by separate plasmids [29]. In this safer system, Wu, et al. showed that the lentiviral packaging functions could be supplied from separate plasmids, although they did not truly physically split the Gag-Pol gene. The Gag-PR plasmid they used had a stop codon at the start of RT and IN to prevent the expression of RT and IN, but the RT and IN sequences remained as part of their Gag-PR expression plasmid. This configuration was exposing to residual risk of RCL formation by sequence read-through, reversion or recombination. In contrast, the split packaging systems presented here establish the functionality of creating a true physical split of the Gag-Pol gene, where neither Gag-PR nor Gag expression plasmids contains RT or IN sequences. Tat and Rev are also provided from completely separated expression plasmids.

In our first attempt at constructing this split-packaging system, the Gag-Pol polyproteins were expressed using two expression plasmids: one for Gag-PR and the second expressing RT/IN. As was shown in the Results, this first generation system (the 6 plasmid system) produces infectious viral particles at titers 9 fold lower than those generated by the conventional lentiviral packaging system in which Gag-Pol is supplied intact from a single expression plasmid. After examining the profile of viral proteins from virions produced by the 6 plasmid system, we determined that RT/IN was not efficiently processed although it was incorporated into the viral particles. The same phenomenon was observed for the Gag p55 precursor. Because PR is central to processing the precursor polyproteins, the reduced processing of Gag and RT/IN suggested that the low titers might be explained by a defect in the activation and release of PR. Another contributing factor that could explain the low titers is the accumulation of uncleaved Vpr-RT/IN fusion proteins. We have previously shown that incorporation of Vpr fused heterologous amino-acid sequence affected the infectivity of HIV-1 viral particles [27].

To improve upon this first generation split-packaging system, we then developed a new "super-split" system (the 7 plasmid system) in which Gag is not only separated from Pol, but PR is separated from Gag and supplied independently *in trans*. It was our hope that, by supplying PR *in trans*, we could increase the amount of active PR and improve processing of the precursor proteins. This approach raised two theoretical concerns: the potentially enhanced cytotoxic effect of PR [9,14,17] and the possible premature processing of the precursor polyproteins [5,14,17]. To address the issue of cytotoxicity, we used a mutant PR with slightly reduced protease activity and none of the cytotoxic effects seen with the *wt *PR [38]. We found that supplying PR *in trans *as part of the 7 plasmid system resulted in titer improvement comparatively to those obtained with the 6 plasmid system. Furthermore, the mutant PR supplied *in trans *yielded viral titers higher than those obtained with the use of *wt *PR. A concurrent improvement upon processing of both the Pr55Gag and RT/IN polyproteins was also observed. When the 7 plasmid system was compared to the conventional lentiviral packaging system, the viral titers were only 3 fold lower with a mean titer of 1.0 × 10^6 ^TU/ml for unconcentrated virus. In addition to the data presented here and to demonstrate that the viral particles generated by the 7 plasmid system can be concentrated and used to transduce dividing and non-dividing cells, the efficacy of this "super-split" packaging system with the PR supplied *in trans *was demonstrated by us in a published study where the 7 plasmid system was used to transduce human cord blood hematopoietic stem cells with a complex beta-globin expressing lentiviral vector assessed in NOD-SCID mouse transplant studies [40].

In order to generate the safest possible lentiviral packaging system, we incorporated several safety features into the 7-plasmid system. These features include: (i) splitting the Gag, PR, and RT/IN functions into separate plasmids, (ii) eliminating the frameshift, so that even in the event of a recombination event, Pol could not be produced, and (iii) minimizing the overlapping sequences that existed between the lentiviral vector (packaging signal) and the Gag expression plasmid (from 542 to 55 bases), and between the Gag and Pol (from 208 to 54 bases). In addition to the packaging systems, we also compared viral titers produced by the *wt*-LTR and SIN lentiviral vectors and found that the SIN vector produced equivalent or higher viral titers for all three packaging systems, possibly due to the benefit conferred by the "ideal" poly(A) sequence that we substituted for U5 within the 5' LTR. Iwakuma, et al., also reported an increase in viral titers when they replaced the U5 region of their SIN vector with a bGH poly(A) sequence [41]. Most importantly, this increase resulted in viral titers for the 7 plasmid system in conjunction with the SIN vector that were only 2 fold lower than those obtained by the conventional system using a *wt*-LTR vector, 1.0 × 10^6 ^TU/ml and 2.2 × 10^6 ^TU/ml, respectively.

## Conclusion

Here we presented a novel "super-split" lentiviral packaging system with the potential to be used for gene therapy applications. Since this system incorporates many new safety features while using a less cytotoxic mutant PR, it also presents new opportunities to develop better high titer HIV packaging cell lines.

## Methods

### Plasmid construction of transfer vectors

All the lentiviral components used in plasmid construction with the exception of Vpr [27] and RRE [42], were derived from PLNENV-1, accession # M19921 [43]. Oligonucleotides used were purchased from Life Technologies and all PCR products were verified by sequencing. The basic vector design of the *wt*-LTR and SIN lentiviral vectors has been described previously [44]. Briefly, both of the *wt*-LTR and SIN vectors contain a packaging signal, central polypurine tract, RRE (Rev response element), and an Ef1α promoter [45] driving the expression of eGFP (Clontech), which replaces the β-Globin cassette located between the BamHI and Kpn I restriction sites. The SIN vector contains a 400 bp deletion between EcoRV and Pvu II sites in the U3 region of the 5' LTR as well as a complete deletion of the U5, which was replaced by an "ideal" termination/polyadenylation sequence (ATG TGT GTG TTG GTT TTT TGT GT). In addition the SIN vector also contains two stops in the packaging signal placed at the 1^st ^and 35^th ^amino acid of Gag to prevent translation of Gag.

### Plasmid construction of Gag expression plasmids

The backbone used for all three Gag expression plasmids, Gag-Pol-Vif, Gag-PR, and Gag alone, was "pCI Vector" (Promega) in which Nhe I site was replaced by a BssHII linker, the CMV promoter from the Bgl II to the Sac I sites was replaced by a Nhe 1 to Sac I fragment from cRev plasmid [42], this inserted the SV40 origin next to CMV promoter, and finally a RRE obtained from the Bgl II to Hind III sites of pgTatCMV [42] was inserted by blunt ligation into the Xba I to Sal I sites of the pCI Vector backbone. The Gag-Pol-Vif plasmid was made from digesting the backbone with BssHII and EcoRI and then inserting a 5032 bp BssHII to EcoRI Gag-Pol-Vif fragment from PNLENV-1. The Gag-PR plasmid was made by digesting the Gag-Pol-Vif plasmid with Bgl II and EcoRI to remove Pol-Vif, and then PCR was used to create a 463 bp fragment containing PR with a stop and EcoRI site. The primers used for PCR are 5' GGG A**AG ATC T**GG CCT TCC CAC 3' and 5' CG**G AAT TC**G GAT CC**T TA**A AAA TTT AAA GTG CAG CCA ATC TGA CT 3'. The Gag plasmid was made by first replacing the fragment from Nsi I to Bgl II with a PCR version (845 bp), in which the frameshift had been altered. The primers used are 5' TAA **ATG CAT **GGG TAA AAG TAG TA 3' and 5' CC**A GAT CT**T CCC **TAA GAA G**TT AGC CTG TCT CTC AGT ACA ATC 3'. Next the Bgl II to EcoRI fragment was replaced by a 208 bp PCR product, which contained P6 with an EcoRI site placed after the stop (all Pol components were removed). The primers used for PCR are 5' GGG A**AG ATC T**GG CCT TCC CAC 3' and 5' CG**G AAT TC**G CTA GCT ATC TTT ATT GTG ACG AGG GGT C 3'. The optimized version of the Gag plasmid was created by altering the coding sequence for the CAp24 and the start of MAp17 (502 bp), from BssHII to Nsi I sites, using PCR to first anneal 26 overlapping 40 mer oligonucleotides (sequence available upon request) and then a second PCR to create the 502 bp BssHII to Nsi I fragment from the annealed oligonucleotides. This protocol was described previously in [46,47].

### Plasmid construction of VPR fusion plasmids

The backbone used to construct the Vpr fusion plasmids was "pCI Vector" (Promega) in which the CMV promoter from Bgl II to Mlu I had been replaced with an Hpa I to Mlu I fragment containing the Ef1α promoter [45]. The Vpr-RT/IN-Vif fusion plasmid was made by first inserting the 1–88 truncated Vpr (276 bp) into the Xba I site of the pCI Vector backbone. The Bgl II site at the end of Vpr was ligated to a Bgl II to EcoRV 468 bp PCR fragment consisting of the start of RT, keeping RT in frame with Vpr and the PR cleavage site intact. The primers used for PCR were 5' GGA **AGA TCT**CTG TTG ACT CAG ATT G 3' and 5' GTA CT**G ATA TC**T AAT CCC TGG 3'. The EcoRV site from the PCR fragment was ligated to an EcoRV to Not I 3372 bp fragment from the Gag-Pol-Vif plasmid containing the rest of RT, IN, Vif, and the RRE. Finally, in the Vpr-RT/IN-Vif plasmid a splice donor site was placed before Vpr (to ensure proper expression of Vif) using annealed oligonucleotides spanning the Mlu I to Xba I sites. The annealed oligonucleotides used were 5' **CGC GT**G CTA GCG GCG ACT **GGT G**AG TAC GCC A**T **3' and 5' **CTA GA**T GGC GTA CT**C ACC**AGT CGC CGC TAG C**A **3'. The Vpr-PR plasmid was made from the Vpr-RT/IN-Vif plasmid by digesting this backbone with Bgl II and EcoRI and then ligating the annealed oligonucleotides from Bgl II to BspEI and a 231 bp BspEI to EcoRI PCR fragment generated from the Gag-PR plasmid. The annealed oligonucleotides consisted of 5' **GAT CT**G TAT CCT TTA GCT TCC CTC AGA TCA CTC TTT GGC AGC GA 3', 5' CCC CTC GTC ACA ATA AAG ATA GGG GGG CAA TTA AAG GAA GCT CTA TTA GAT **T **3', 5' **CCG GA**A TCT AAT AGA GCT TCC TTT AAT TGC CCC CCT ATC TTT ATT GTG ACG A 3', 5' GGG GTC GCT GCC AAA GAG TGA TCT GAG GGA AGC TAA AGG ATA C**A **3' and were used to fuse Vpr in frame to PR at the Bgl II site while maintaining the PR cleavage site and inserting a T26S mutation (ACA changed to TCC) thereby creating a BspEI site. The primers used to PCR the 5' portion of PR and to create a stop at the end of PR are 5' GAA GAT CTA CGC GT**T CCG GA**G CAG ATG ATA CAG TAT TAG AAG 3' and 5' CG**G AAT TC**G GAT CC**T TA**A AAA TTT AAA GTG CAG CCA ATC TGA GT 3'.

### Cells

Human embryonic kidney (HEK) 293T cells were maintained in Dulbecco's modified Eagle's medium (DMEM) supplemented with 8% bovine growth serum, 2% fetal calf serum and 100-units/ml penicillin-streptomycin. NIH 3T3 were maintained in DMEM media supplemented with 10% calf serum and 100-units/ml penicillin-streptomycin. Jurkat cells were maintained in RPMI media supplemented with 10% fetal calf serum and 100-units/ml penicillin-streptomycin.

### Virus production and titers

Virus was produced by transient transfection of 293T cells (10 cm dish) using Fugene (Roche). Twenty-four hours prior to transfection 293T cells were split 1:4 and at two hours prior to transfection media was removed and fresh media was added. Transfections were done using 5, 6, or 7 plasmids depending on the packaging system used. Plasmids transfected for the 5 plasmid system consisted of 3.2 μg lentiviral vector (*wt*-LTR or SIN, expressing GFP), 4 μg Gag-Pol-Vif packaging plasmid, 0.4 μg of each Rev, Tat, and VSV-G expression plasmids, for the 6 plasmid system the Gag-Pol-Vif plasmid was replaced by two plasmids one expressing Gag-PR (5.5 μg) and the other Vpr-RT/IN-Vif (2.0 μg), and for the 7 plasmid system the Gag-Pol-Vif plasmid was replaced by three plasmids expressing Gag (4.0 μg), Vpr-PR (1.8 μg) and Vpr-RT/IN-Vif (1.8 μg). Supernatants were collected and filtered through a 0.45 μM filter 48 h after transfection. Titers were determined by infecting either NIH 3T3 cells (2 × 10^5 ^cells/6 well dish) or Jurkat cells (3 × 10^6 ^cells/6 well dish) with serial dilutions of viral supernatants in a total volume of 900 μl in the presence of polybrene (8 μg/ml) or protamine sulfate (6 μg/ml), respectively. Transduced NIH 3T3 and Jurkat cells were analyzed, three or more days after infection for the expression of GFP by FACS. Transducing units (TU) were determined by multiplying the number of total cells at the time of infection by the percentage GFP positive cells by the dilution factor.

### Immunoprecipitation analysis

Forty-eight hours post-transfection, cells were washed with PBS and radiolabeled with 250 μCi of [^35^S] methionine per 10 cm dish (Trans^35^S-Label; ICN, Irvine, CA) for 12 h. Following labeling, cell culture supernatants were collected and centrifuged at 3000 rpm for 30 min to remove any remaining cells or cell debris. Labeled viral particles in supernatants were isolated by ultracentrifugation through a 20% sucrose cushion at 32,000 rpm for 2 h using a Beckman Ti 61 rotor. Pelleted viral particles were then lysed in RIPA buffer (10 mM Tris-HCl (pH 7.4), 1 mM EDTA, 100 mM NaCl, 1% Triton X-100, 0.1% SDS, 0.25% sodium deoxycholate and 0.2% phenyl-methylsulfonyl fluoride (PMSF) and immunoprecipitated with the anti-HIV-1 serum (162) as described previously (53). Immunocomplexes were separated by SDS-12.5% polyacrylamide gel electrophoresis and analyzed by autoradiography. Densitometric analysis of autoradiograms was performed with a Molecular Dynamics Personal densitometer using the ImageQuant™ software version 3.22.

## List of abbreviations

HIV-1, Human Immunodeficiency Virus Type 1; Pr55Gag, Gag precursor polyprotein, MAp17, matrix protein; CAp24, capsid protein, NCp7, nucleocapsid protein; PR, protease; RT, reverse transcriptase, IN, integrase; RCR, replication competent retrovirus; RCL, replication competent lentivirus; SIN, self-inactivating; GFP, green fluorescent protein; TU, transducing units

## Competing interests

Among co-authors, K.A.W. and P.L. are minority stockholders with consulting fee from Genetix Pharmaceuticals.

## Authors' contributions

K.A.W. was responsible for the design, cloning, testing, and writing of the manuscript. Z.A. and E.A.C. were responsible for the immunoprecipitation experiments, protein analysis, and contributed to writing the manuscript. P.L. was responsible for the design and writing of the manuscript.
